# The Potential of LPS-Binding Protein to Reverse Amyloid Formation in Plasma Fibrin of Individuals With Alzheimer-Type Dementia

**DOI:** 10.3389/fnagi.2018.00257

**Published:** 2018-08-22

**Authors:** Etheresia Pretorius, Janette Bester, Martin J. Page, Douglas B. Kell

**Affiliations:** ^1^Department of Physiological Sciences, Faculty of Science, Stellenbosch University, Stellenbosch, South Africa; ^2^Department of Physiology, Faculty of Health Sciences, University of Pretoria, Pretoria, South Africa; ^3^School of Chemistry, The University of Manchester, Manchester, United Kingdom; ^4^The Manchester Institute of Biotechnology, The University of Manchester, Manchester, United Kingdom

**Keywords:** Alzheimer-type dementia, amyloid, clotting, dormancy, infection, microbes

## Abstract

Many studies indicate that there is a (mainly dormant) microbial component in the progressive development of Alzheimer-type dementias (ADs); and that in the case of Gram-negative organisms, a chief culprit might be the shedding of the highly inflammagenic lipopolysaccharide (LPS) from their cell walls. We have recently shown that a highly sensitive assay for the presence of free LPS [added to platelet poor plasma (PPP)] lies in its ability (in healthy individuals) to induce blood to clot into an amyloid form. This may be observed in a SEM or in a confocal microscope when suitable amyloid stains (such as thioflavin T) are added. This process could be inhibited by human lipopolysaccharide-binding protein (LBP). In the current paper, we show using scanning electron microscopy and confocal microscopy with amyloid markers, that PPP taken from individuals with AD exhibits considerable amyloid structure when clotting is initiated with thrombin but without added LPS. Furthermore, we could show that this amyloid structure may be reversed by the addition of very small amounts of LBP. This provides further evidence for a role of microbes and their inflammagenic cell wall products and that these products may be involved in pathological clotting in individuals with AD.

## Introduction

The progression of AD is accompanied by a great many observable changes, both molecular and physiological, and it is the commonest form of dementia (Takizawa et al., 2015). It is currently estimated that 5.4 million Americans have Alzheimer’s Disease and that by mid-century the number of people living with Alzheimer’s Disease in the United States alone is projected to grow to 13.8 million (Alzheimers Association, 2016). AD is not only recognized as a neuro-inflammatory but also a systemic inflammatory condition, as AD individuals present with abnormal clotting (hypercoagulation), decreased fibrinolysis (hypofibrinolysis), elevated levels of coagulation factors, hyperactivated platelets, and vascular defects that include cerebrovascular dysfunction, decreased cerebral blood flow, and blood–brain barrier (BBB) disruption (Ripollés Piquer et al., 2004; Lee et al., 2008; Cortes-Canteli et al., 2012; Bester et al., 2015; Maiese, 2015; Nielsen et al., 2015; von Bernhardi et al., 2015; Pretorius et al., 2016a).

**FIGURE 1:**
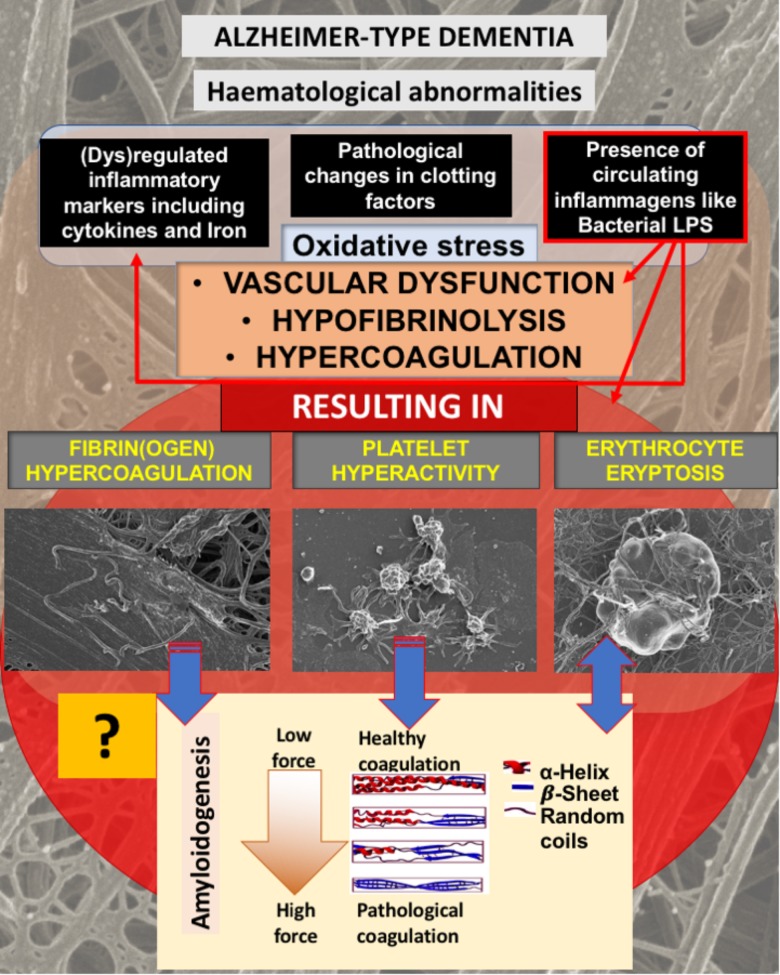
Alzheimer-type dementia (AD) is associated with hematological abnormalities that include (dys)regulated cytokines, iron and clotting factors. Increased LPS levels are also known to be present in AD. We have suggested that the presence of LPS not only is one of the causes of (dys)regulated cytokines, clotting factors and oxidative stress, but the cause of fibrin(ogen) and RBC dysfunction. We investigate here if fibrin(ogen) in AD is amyloid in nature, and if LBP can reverse fibrin(ogen) amyloid structure.

We have previously shown that AD individuals us hematological abnormalities in terms of fibrin(ogen), platelet, and erythrocyte (RBC) structure, and this is summarized in **Figure 1**. In brief, AD individuals exhibit pathological levels of circulating cytokines, and “free” iron levels (albeit typically observed as serum ferritin) are also raised (Kell, 2009; Bester et al., 2013; Kell and Pretorius, 2014; Pretorius and Kell, 2014; Pretorius et al., 2016a). These circulating molecules are known to cause both hypercoagulation and hypofibrinolysis (Kell and Pretorius, 2015b). We have also suggested that, at least in part, the upregulation of cytokines and coagulation factors are due to the presence of potent circulating bacterial cell wall products, that include LPSs (Pretorius et al., 2016a). This purposely implies (as reviewed in Kell and Pretorius, 2018) that many of the pathologies seen in AD are due to the presence of the very potent circulating LPS inflammagen molecules (and other such molecules, e.g., lipoteichoic acid from Gram-positive bacteria). The presence of some sort of infection, with the infectious agents typically in a dormant state (Kell and Pretorius, 2015a; Kell et al., 2015; Potgieter et al., 2015), is central to this line of thought. It is supported by a great many papers that suggest that, although various risk factors have been identified and implicated in AD pathogenesis, including family history and genetics, central to the development of AD is in fact the presence of infections (e.g., Ripollés Piquer et al., 2004; Kamer et al., 2008a,b; Miklossy, 2008, 2011a,b; Honjo et al., 2009; Eriksson et al., 2011; Itzhaki and Wozniak, 2012; Amor et al., 2013; de Souza Rolim et al., 2014; Itzhaki, 2014; Karim et al., 2014; Shaik et al., 2014; Singhal et al., 2014; Singhrao et al., 2014; Gaur and Agnihotri, 2015; Itzhaki et al., 2016).

We recently reviewed the evidence that dormant, non-growing bacteria are a crucial feature of AD, that their growth *in vivo* is normally limited by a lack of free iron, and that it is this iron dysregulation that is an important factor in their resuscitation (Potgieter et al., 2015; Pretorius et al., 2016a; Kell and Pretorius, 2018). We have also presented evidence that bacterial cells can be observed by ultrastructural microscopy in the blood of AD patients (Pretorius et al., 2016a). A consequence of this is that these bacterial cells might shed highly inflammatory components such as LPS. LPS is known to be able to induce (apoptotic, ferroptotic, and pyroptotic; Dong et al., 2015) neuronal cell death. LPS is also raised in AD, and it is found inside the brain and closely associated with the amyloid areas in the brains of these individuals (Lee et al., 2008; Deng et al., 2014; Zhao and Lukiw, 2015; Zhao et al., 2015). Recently, Zhan and co-workers also reviewed literature showing that Gram-negative bacteria (*E. coli*) can induce the formation of extracellular amyloid, and that the degraded myelin basic protein (dMBP) co-localizes with β amyloid (Aβ) and LPS in amyloid plaques in AD brains (Zhan et al., 2018).

We recently also provided evidence that LPS (and LTA from Gram-positive bacteria) could induce amyloid formation in healthy fibrin(ogen), the most abundant plasma protein in blood, after it is added at tiny concentrations to blood from healthy individuals (followed by the clotting agent thrombin) (Pretorius et al., 2016b, 2018a). We then studied the presence of amyloid in these clots (before and after addition of LPS), using confocal microscopy and fluorescent markers for amyloid. In those experiments, we saw that addition of LPS to healthy PPP caused a significant increase of amyloid fluorescent signal, compared to the naïve sample (i.e., samples without added LPS). In these papers, we also showed that LBP can inhibit the formation of such amyloid structures (Pretorius et al., 2016b, 2018a). Furthermore, we showed that (some) of the (naïve) fibrin(ogen) molecules are amyloid in conditions such as type 2 diabetes and Parkinson’s Disease, and that in these conditions, LBP added to PPP of such individuals, could also reduce the extent of amyloid fibrin(ogen) structure (Pretorius et al., 2017a,b, 2018a,b).

Thus, the question now arose as to whether the extent of fibrin-type amyloid in PPP varies between AD individuals and suitably matched controls, and whether the removal of any LPS using the mopping agent, LBP, could remove the amyloid signal present in the (naïve) plasma of AD individuals.

Indeed, Zhang et al. (2009) reported elevated levels of LPS concentrations in plasma from patients with sporadic amyotrophic lateral sclerosis and AD, as compared to healthy controls. The present paper provides further evidence of the presence of LPS in PPP of AD individuals, as we showed that LBP could remove amyloid (fluorescent) signal from AD plasma. Our observation is therefore consistent with the general view set out above that there is a major dormant microbial component to AD.

## Materials and Methods

### Ethical Statement, Volunteer Details, and Blood Collection

Blood samples were obtained from non-smoking, Alzheimer-type dementia (AD) patients, identified by a Neurologist and under the care of a medical practitioner. Specifically, care was taken to exclude vascular dementia. We also recruited “healthy” age-matched individuals that did not smoke. It should be noted that the term “healthy” is used in this paper to describe an individual who does not have dementia. Ethical clearance was obtained from the Health Sciences Ethical committee from the University of Pretoria, and informed consent was obtained from family members who act as carers of the patients (81/2013, amended 2015). Healthy individuals also filled in consent forms. Blood was collected in two 4 mL citrate tubes and one 4 mL clotting tube for iron level determination. This collection and all handling of samples were performed under very strictly aseptic conditions, to prevent any microbial contamination of samples.

### Iron Tests

Serum ferritin, transferrin, and serum iron was tested at a pathology laboratory in South Africa.

### LPS-Binding Protein

A final added LBP exposure concentration of 4 ng L^-1^ LBP was used and LBP was purchased from Sigma (recombinant product SRP6033; >95% pure).

### Scanning Electron Microscopy (SEM) of Platelet Poor Plasma (PPP)

At least 30 min after the blood was collected in citrate tubes by venepuncture, PPP were obtained and frozen at -80°C. PPP was prepared by centrifuging citrated whole blood for 15 min at 3,000 *g* at room temperature. After all samples were collected, PPP were thawed and 10 μL mixed with 5 μL thrombin to create an extensive fibrin network. Thrombin was provided by the South African National Blood Service, and the thrombin solution was at a final exposure concentration of 10 U mL^-1^ (initial product concentration is 20 U mL^-1^ made up in PBS containing 0.2% human serum albumin, see footnote 1 for a description of how thrombin units are calculated). A Zeiss ULTRA Plus FEG-SEM with InLens capabilities was used to study the surface morphology of erythrocytes, and micrographs were taken at 1 kV. SEM preparation was done as previously reported (Pretorius et al., 2017c).

### Airyscan Confocal Microscopy

PPP was thawed, followed by preparation of clots for analysis using confocal Airyscan methods. We added Thioflavin T (ThT) (a well-established amyloid stain; LeVine, 1999; Biancalana et al., 2009; Biancalana and Koide, 2010; Groenning, 2010; Sulatskaya et al., 2011, 2012; Kuznetsova et al., 2012; Picken and Herrera, 2012; Younan and Viles, 2015; Kuznetsova et al., 2016; Rybicka et al., 2016) at a final concentration of 5 M to 200 μL to either healthy PPP, naïve AD PPP, or after a 10 min exposure of AD PPP to 4 ng L^-1^ (final concentration) LBP. These PPP samples were incubated (protected from light) for 1 min. This step was followed with the addition of thrombin, added in the ratio 1:2 to create extensive fibrin networks. A coverslip was placed over the prepared clot, and viewed immediately with a Zeiss LSM 510 META confocal microscope with super-resolution (Airyscan) capabilities. The Airyscan detector increases the resolution by a factor of 1.7, achieving super-resolution of 140 nm, and with a Plan-Apochromat 63×/1.4 Oil DIC objective. Excitation was at 488 nm and emitted light was measured at 505–550 nm.

### Statistical Analysis and Data-Sharing

#### Histogram-Based Analysis of SEM and ThT Staining

For each picture, we obtained the histogram of intensities (8-bit scale) using the *histogram* function of ImageJ. From this we calculated the coefficient of variation (CV; as standard deviation/mean). For details of this analysis method, see (Pretorius et al., 2017b, 2018a). Quantification of fluorescent marker binding (ThT) was done by assessing the variance between (black) background and the presence of fluorescent pixels where ThT fluorescent binding was present in the clots. Increased ThT binding is here reflected as increased fluorescence which shows increased amyloid protein structure in fibrin(ogen) (see Pretorius et al., 2017a, 2018a) for a detailed explanation of the methods. We used the histogram function in ImageJ (FIJI) and calculated the coefficient of variation (CV) (as SD/mean) of the histogram of different pixel intensities as our metric to quantify and discriminate between clots of healthy (age-controlled) naïve PPP and clots from AD with and without LBP.

A healthy clot (i.e., a clot taken from a healthy individual), viewed with SEM looks somewhat like a bowl of spaghetti with elongated fibrin fibers. In AD individuals, this clot structure changes to a dense and matted hypercoagulated clot (Bester et al., 2015). We also used the CV calculation described above to analyze SEM clots. The fibrin fibers of healthy individuals have a greater variation of dark and light areas, due to the elongated fibers, with open areas between the individual fibers. With an increased hypercoagulability and amyloid formation, the clots become matted and dense, resulting in a more uniform grayness. We used this difference in structure as our metric, where increased hypercoagulability is related to an increase in amyloid formation and this is visible as a more uniformly dense morphology with less color gradient.

The statistical analysis of CV data was performed with GraphPad 7, using the one-way ANOVA analysis with Tukey’s multiple comparison’s test comparing the mean of each column with the mean of every other column.

#### Availability of Data and Material

Raw data, including original micrographs can be accessed at: https://1drv.ms/f/s!AgoCOmY3bkKHiJRrop6cF6uhTnQA1A or https://www.researchgate.net/profile/Etheresia_Pretorius.

## Results

As discussed in the introduction, AD is not only known for the presence of neuroinflammation, but also for the presence of hematological abnormalities, including an increased presence of LPS and also (dysregulated) cytokines, iron and clotting factors, which result in oxidative stress and abnormal clotting. Previously we showed that abnormal clotting and the presence of bacterial inflammagens like LPS, result in fibrin(ogen) becoming amyloid in nature, and that we can remove the signal by addition of LBP (Pretorius et al., 2017a,b, 2018b). In **Figure 1**, we set out our hypothesis: that also in AD, the presence of LPS, together with dysregulated iron levels and oxidative stress, causes fibrin(ogen) to become amyloid and that we can reverse this with LBP. Furthermore, we show this reversal by using both ultrastructure (SEM) and the fluorescent marker ThT using Airyscan (confocal) microscopy. The rationale behind using LBP is that, if the amyloid structure is indeed due to the presence of bacterial inflammagens, LBP would remove it by binding to these inflammagnes, thus preventing it from causing amyloid fibrin(ogen) deposits.

**Table 1** shows the demographics of individuals with AD, as well as healthy, age-controlled individuals. Transferrin, iron, % saturation of iron and serum ferritin were measured in these individuals, and these values, particularly serum ferritin, is used as an indication of the level of systemic inflammation (Kell, 2009; Kell and Pretorius, 2014).

**Table 1:** Demographics for the healthy and the Alzheimer-type dementia individuals used in this study.

	Alzheimer’s disease (*N* = 20)	Healthy individuals (*N* = 11)	*p*-Values
Gender	15 F; 5 M	7 F; 4 M	0.7
Age	77.3 ± 12.1	70.0 ± 13.0	0.13
Iron (μM)	12.4 ± 5.02	19.0 ± 4.39	**0.001**
Transferrin (g⋅L^-1^)	2.2 ± 0.47	2.4 ± 0.30	0.13
% transferrin saturation	24.2 ± 10.79	31.9 ± 7.52	**0.04**
Serum ferritin (ng⋅mL^-1^)	96 (30.5–113)	66 (29–84)	0.4


*Gender was compared using Fisher’s exact test. Age and iron measurements were compared using the unpaired *t*-test. Serum ferritin was compared using the Mann–Whitney test following the non-normal distribution of this measurement. All analyses were done using GraphPad 7. Data are presented as either mean ± STD or median (lower quartile–upper quartile; interquartile range). Bold numbers show significant *p*-values.*

In our hypothesis and **Figure 1** we argue that there is a link between oxidative stress, increased iron levels and inflammation, and this is directly linked to the presence of bacterial inflammagens like LPS. In our sample, healthy individuals had low mean serum ferritin, where in the AD population it was approximately three times higher. However, despite the large difference in mean serum ferritin values between the two groups, the difference was not statistically significant owing to large variation within the samples.

**Table 2** shows results for the analysis of the clots using both SEM and confocal microscopy. Micrographs were analyzed as discussed in Section “Materials and Methods”. **Table 2** shows *p-*values and statistics of CVs calculated from SEM (micrographs showing ultrastructure) and Airyscan (micrographs showing fluorescence). We compared CVs from controls and AD individuals, and that produced the *p*-values (**Table 2**).

**Table 2:** Data for Alzheimer-type dementia and healthy individuals showing the coefficients of variation (CV) of the intensity of the pixels in the clot images (Tukey’s analysis).

	*p*-Value	Mean difference	95.00% CI of difference
**Airyscan coefficients of variation *p*-values (AD: *N* = 20; Control: *N* = 10)**		
Control vs. AD	**<0.0001**	-0.35	-0.5 to -0.2
Control vs. AD + LBP	0.8	0.05	-0.14 to 0.2
AD vs. AD + LBP	**<0.0001**	0.39	0.2 to 0.5
**Scanning electron microscopy coefficients of variation *p*-values (AD: *N* = 20; Control: *N* = 11)**		
Control vs. AD	**<0.0001**	0.2	0.1 to 0.3
Control vs. AD + LBP	0.06	-0.07	-0.15 to 0.003
AD vs. AD + LBP	**<0.0001**	-0.3	-0.4 to -0.24


*Airyscan and SEM images were used and statistical analysis was done to compare CVs from controls vs. AD individuals. Bold numbers show significant *p*-values.*

**FIGURE 2:**
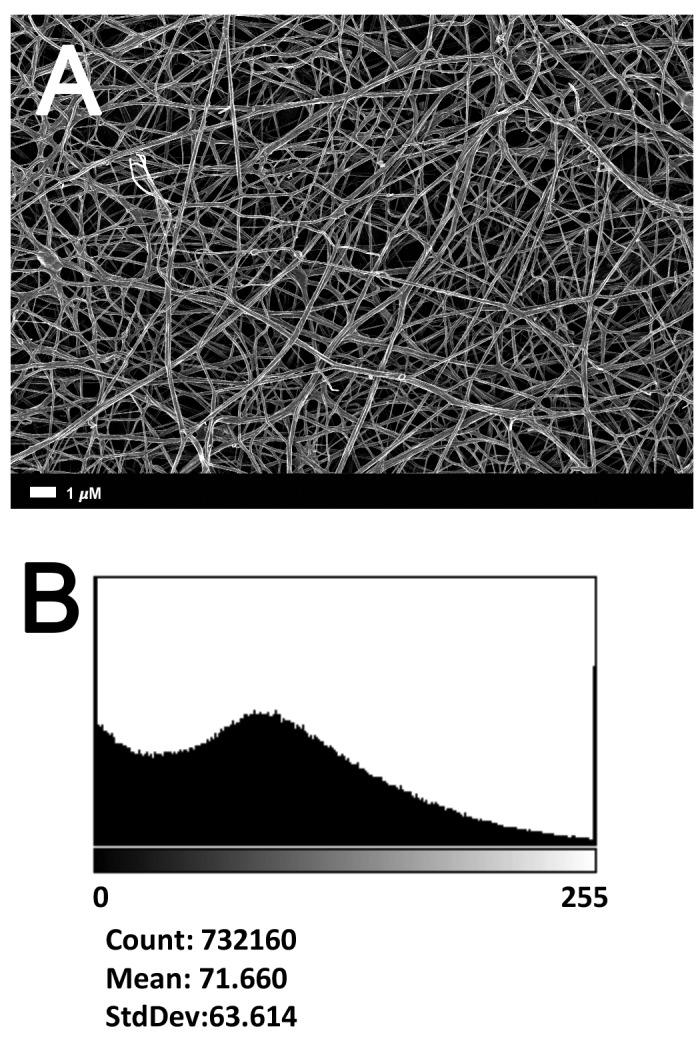
**(A)** Clot structure from a representative healthy individual as seen with SEM. All clots were created by adding thrombin to PPP (prepared after whole blood is centrifuged for 15 min at 3,000 *g*). **(B)** Representative histogram of the 8-bit intensity for the SEM clot shown in **(A)**.

**FIGURE 3:**
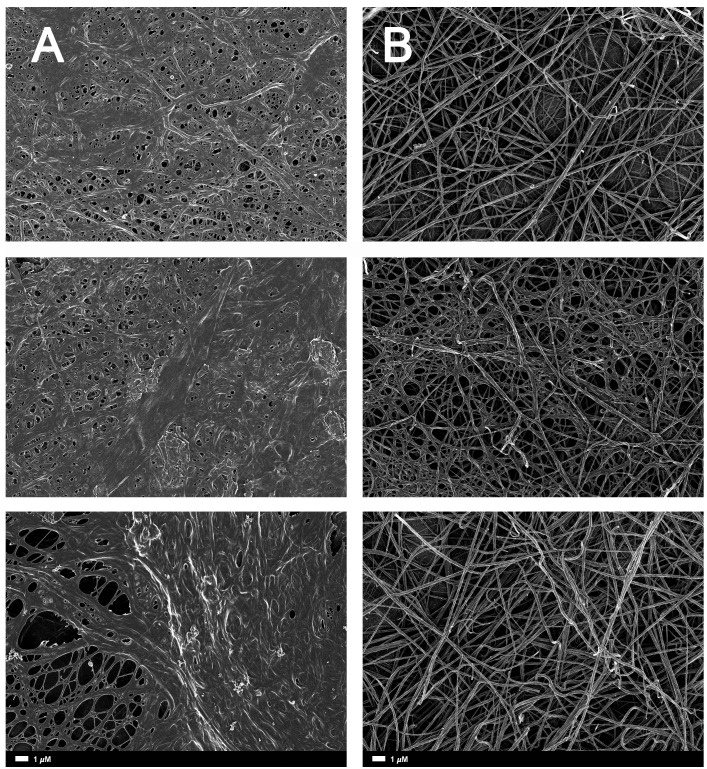
**(A)** Naïve clot structures from representative Alzheimer-type dementia individuals as seen with SEM. **(B)** The same samples treated with LBP. The size marker is the same for all panels.

**Figure 2A** gives an example of the clot structure, as viewed with SEM, from a representative healthy individual. We analyzed each SEM micrograph with ImageJ and produced a histogram that gave us the mean and the standard deviation for each micrograph (see section “Materials and Methods”). **Figure 2B** shows such a representative histogram of the 8-bit intensity for the SEM micrograph shown in **Figure 2A**. All micrograph histograms were used to calculate the CVs for each participant (both controls and AD individuals) (statistical analysis shown in **Table 2**). **Figure 3** shows SEM images before and after treatment of a representative examples of three AD PPP clots, with and without LBP.

**FIGURE 4:**
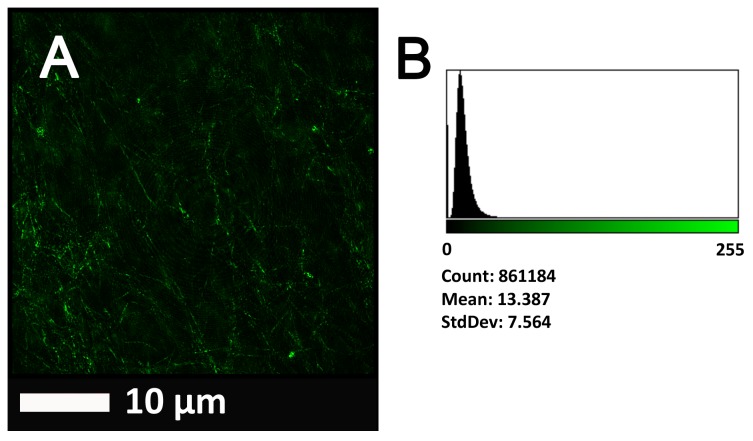
**(A)** Clot structure from a representative healthy individual as seen with Airyscan super-resolution confocal microscopy. PPP from each individual was incubated with the fluorescent marker ThT. PPP were mixed with thrombin to create an extensive fibrin network. **(B)** Representative histogram of the 8-bit intensity for the Airyscan clot shown in **(A)**.

**FIGURE 5:**
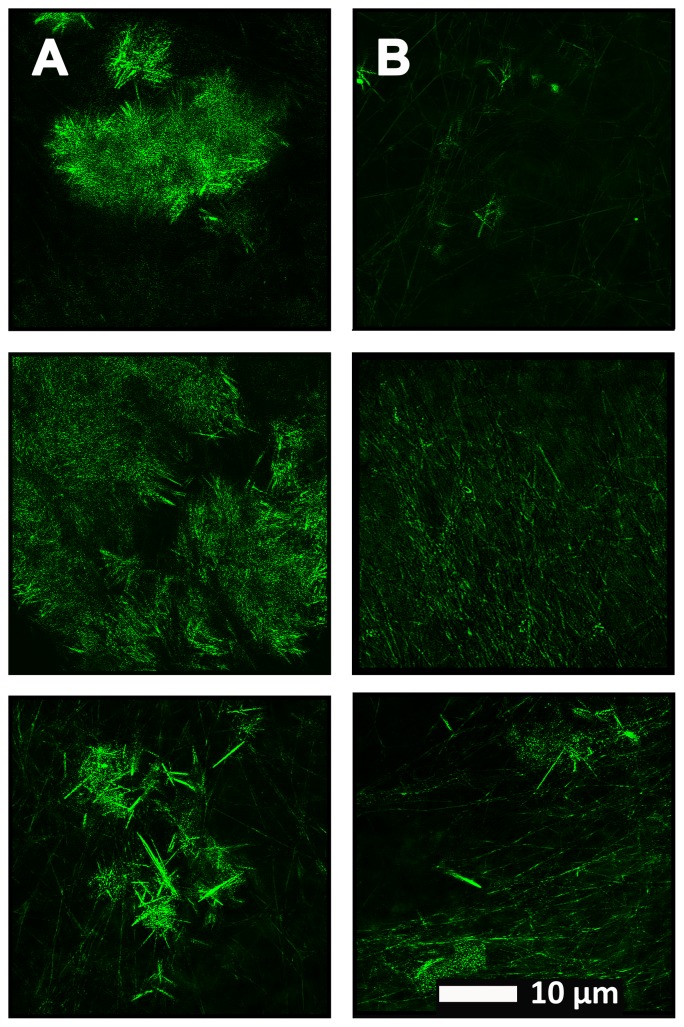
**(A)** Naïve clot structure from representative Alzheimer-type dementia individuals as seen with Airyscan super-resolution confocal microscopy. PPP from each individual was incubated with the fluorescent marker ThT. PPP were mixed with thrombin to create an extensive fibrin network. **(B)** Micrograph of the PPP clots from the same individual in the opposite column **(A)**, after treatment with LBP, followed by addition of ThT and clot preparation.

**FIGURE 6:**
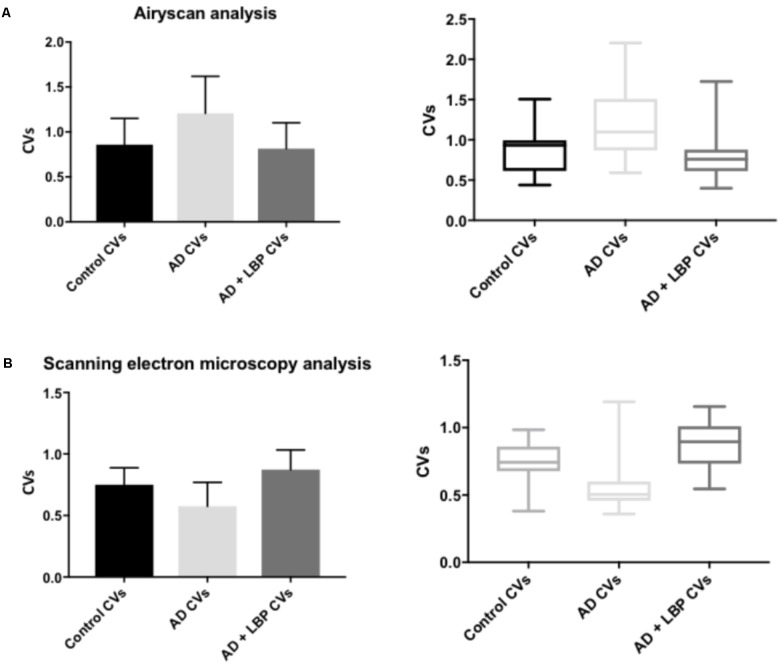
Graphs and boxplots from coefficient of variation (CVs) form histogram data of Airyscan analysis **(A)** and SEM **(B).** Coefficients of variation (CV) of the intensity of the pixels in the clot images was done using the Tukey’s analysis. Control vs. AD and AD vs. AD + LBP significantly differ by < 0.0001. Control vs. AD + LBP are not significantly different.

**Figure 4** show a representative micrograph and its histogram from a healthy individual, using Airyscan confocal microscopy. **Figure 5** shows clots from AD individuals before and after LBP treatment. In healthy clots, there is little to no binding of ThT to amyloid fibrin(ogen) proteins. In AD clots, significant ThT binding fluorescence is noted, suggesting increased amyloid formation in fibrin(ogen). When LBP is added to AD PPP, ThT show significantly decreased binding. **Figures 6A,B** show graphs and boxplots from the CV analysis. LBP added to PPP from AD individuals (with added thrombin to initiate clotting), seems to aid in the removal of amyloid signal so that the fibrin(ogen) structure now looks more like that of the controls (noted by using two techniques: Airyscan and SEM). Furthermore, the *p*-values between controls vs. AD with added LBP in both the Airyscan and SEM analysis, showed that added LBP makes AD clots not significantly different to the controls (*p* = 0.8 and 0.06).

## Discussion

We have previously determined that in many inflammatory conditions, the “normal” clotting of blood, involving the polymerisation of fibrinogen to fibrin, produces a fibrin fiber structure that becomes amyloid in nature, and that this might be due to the presence (in part) of the potent inflammagen LPS, which comes from the membranes of Gram-negative bacteria (Potgieter et al., 2015, 2016b, 2017b, 2018a; Kell and Pretorius, 2017a,b) and is a potent inflammagen (Walter et al., 2007; Kell and Pretorius, 2015a). This would be consistent with the many studies (reviewed in Miklossy, 2015; Itzhaki et al., 2016; Kell and Pretorius, 2018) that imply that there is a (dormant) microbial component in AD. Previous research (see Poole et al., 2013; Bester et al., 2015; Zhan et al., 2016a,b; Zhao et al., 2017a,b) found LPS inside the brains of Alzheimer’s disease patients, as well as an increase in circulating LPS. LPS is known to cross (and possibly to damage Liu et al., 2001; Xaio et al., 2001; Jaeger et al., 2009; Jangula and Murphy, 2013; Banks et al., 2015) the BBB and lead to β-amyloid depositions (Lee et al., 2008). Furthermore, neurotoxic microbial-derived components from the GI tract microbiome can cross aging GI tracts and BBBs and contribute to progressive proinflammatory neurodegeneration (Zhao and Lukiw, 2018). In a recent review, Zhan and co-workers describe that LPS indeed associates with amyloid plaques, neurons and oligodendrocytes in AD brains (Zhan et al., 2018). These authors also showed that LPS infiltrates the AD nucleus and can induce an inflammatory signaling program in brain cells, including up-regulation of the pro-inflammatory microRNA miRNA-146a via a NF-kB signaling circuit (Zhan et al., 2018).

Here we also show that the hypercoagulable structure of fibrin(ogen) in AD patients is different from healthy individuals, in that they appear to be amyloid (as shown with the fluorescent marker ThT) and that their structure, viewed with SEM, is matted and dense. In healthy clots, fibrin has a typical “spaghetti-like” structure (Kell and Pretorius, 2015b). We could reverse aberrant clotting in AD PPP by the addition of LBP. LBP binds bacterial inflammagens and our results would therefore point to the presence of bacterial inflammagens in AD PPP – that is, LBP could bind to and thus prevent these inflammagens from causing amyloid formation in the AD PPP when clots are formed after addition of thrombin.

When we added LBP to PPP from AD individuals (by incubating their PPP with LBP), we showed that the *p*-values were not significantly different (*p* = 0.8 and 0.06) between AD and control donor blood. Therefore, LBP, incorporated in a therapy, might not only prevent aberrant clotting in these individuals, but might also reduce the circulating LPS pool that could eventually cross into their brains via the BBB. Of course, a damaged BBB can admit the transfer (atopobiosis; Potgieter et al., 2015) of the organisms themselves (Miklossy, 2011a, 2015; Bajpai et al., 2014; Tang et al., 2017), where they may be detected ultrastructurally (Mattman, 2001), and that may continue to shed inflammagens. We therefore suggest that LBP might eventually be used as treatment to prevent the damaging effect of LPS on fibrin(ogen) and hypercoagulation, and even to prevent (at least in part) the deposition of amyloid-β (Aβ) plaques in the brain and the loss of cognitive function that accompanies this neurodegenerative disease. However, we note that a control protein, such as human IgG should, in future, be used to present the specific effect of LBP on amyloid formation, to further elucidate the physiological processes discussed in this paper. In future, our hypothesis could also be tested in a transgenic murine model of AD (TgAD) or the 5xFAD (amyloid over-producing) model or equivalent (Vale et al., 2010; Jeong et al., 2018).

## Author Contributions

EP study leader, prepared all the figures, and co-wrote the paper. JB prepared and analyzed all the samples. MP statistical analysis and the paper editing. DK study co-leader, and co-wrote and edited the paper. All authors reviewed the manuscript.

## Conflict of Interest Statement

The authors (DK and EP) declare the following patent application: method for treating Alzheimer’s Disease (P3448ZA00-AS2CA).The remaining authors declare that the research was conducted in the absence of any commercial or financial relationships that could be construed as a potential conflict of interest.

## Footnotes

1 ^1^https://www.sigmaaldrich.com/content/dam/sigma-aldrich/docs/Sigma/Product_Information_Sheet/1/t6884pis.pdf

## Abbreviations

AD: Alzheimer-type dementia
LBP: lipopolysaccharide-binding protein
LPS: lipopolysaccharide
PPP: platelet poor plasma
SEM: scanning electron microscope
